# Soluble Klotho is not independently associated with cardiovascular disease in a population of dialysis patients

**DOI:** 10.1186/1471-2369-15-197

**Published:** 2014-12-11

**Authors:** Maurits S Buiten, Mihály K de Bie, Annet Bouma-de Krijger, Bastiaan van Dam, Friedo W Dekker, J Wouter Jukema, Ton J Rabelink, Joris I Rotmans

**Affiliations:** Department of Cardiology, Leiden University Medical Center (LUMC), Leiden, The Netherlands; Department of Internal Medicine, Spaarne Hospital, Hoofddorp, The Netherlands; Department of Nephrology, MCA, Alkmaar, The Netherlands; Department of Clinical Epidemiology, LUMC, Leiden, The Netherlands; Department of Nephrology, Leiden University Medical Center, P O Box 9600, 2300 RC Leiden, The Netherlands

**Keywords:** End stage renal disease, Dialysis, Klotho, Cardiovascular disease

## Abstract

**Background:**

Dialysis patients suffer from a high burden of cardiovascular disease (CVD). Partly this is due to progressive deterioration of calcium-phosphate homeostasis. Previous studies suggested that besides FGF-23, low levels of Klotho, a protein linked to aging, might constitute a key factor in this detrimental relationship. The purpose of the present study was to determine the relationship between serum Klotho (sKlotho) and the presence of CVD in dialysis patients.

**Methods:**

Plasma levels of sKlotho were measured in a cohort of dialysis patients and related to left ventricular (LV) dysfunction (defined as a LV ejection fraction <45%) and LV mass using echocardiography. Coronary artery disease (CAD) and calcification score were assessed using computed tomography angiography. Abdominal aortic calcification score (AACscore) was measured by abdominal X-ray.

**Results:**

We included 127 dialysis patients, 67 ± 7 years old, 76% male, 67% on hemodialysis, median sKlotho 460 pg/mL (25th-75th percentile 350-620 pg/mL). Patients with a low sKlotho (<460 pg/mL) showed significantly more CAD (81% versus 61%; p = 0.02) and LV dysfunction (19% versus 3%; p < 0.01). However, after adjusting for confounders, sKlotho was not independently associated with the presence of CVD or the AACscore.

**Conclusions:**

In the present cohort of dialysis patients, sKlotho was not independently associated with CVD. However, patients with a low sKlotho level (<460 pg/mL) did show CAD and LV dysfunction more frequently. Therefore, while sKlotho might be a marker for CVD in dialysis patients, the current data does not support a direct cardioprotective effect of sKlotho.

## Background

Patients with chronic kidney disease (CKD) suffer from a high burden of cardiovascular disease (CVD) [1, 2]. Whereas part of this burden is related to traditional risk factors, CKD associated disturbance in calcium-phosphate homeostasis play a crucial role as well [3]. Recently, Fibroblast growth factor-23 (FGF-23) and its co-receptor Klotho have emerged as pivotal players in calcium-phosphate homeostasis and they could be the missing link in the detrimental relationship between CKD and CVD [4].

Membrane-bound Klotho is a co-receptor for FGF-23 vital for FGF-23’s phosphaturic effect. In CKD serum FGF-23 levels rise in response to phosphate retention, while Klotho levels decrease [5]. Elevated FGF-23 levels are associated with worse cardiovascular outcome but it is unknown whether this is due to the toxic effect of FGF-23 itself or due to Klotho deficiency causing a state of FGF-23 resistance. Studies examining the relationship between low Klotho and outcome show conflicting results [6, 7].

Klotho was first identified in mice, where mutations of the gene led to a syndrome resembling aging [8]. The Klotho protein exists in both a membrane bound and a soluble form (sKlotho). sKlotho is derived from the extracellular part of membranous Klotho and through alternative splicing of the Klotho gene and has a unique role in renal calcium and phosphate excretion, independent of FGF-23 [9–12]. Furthermore, sKlotho has been associated with protective effects on vascular calcification and oxidative stress in preclinical studies [13, 14]. Since the kidneys are the principal source of sKlotho, some studies have suggested that CKD might be a state of progressive Klotho deficiency [15, 16].

Given the strong cardioprotective effects of Klotho demonstrated in preclinical studies, the current study aimed to assess the association between sKlotho and CVD in patients on chronic dialysis. It was hypothesized that low levels of sKlotho are associated with a larger burden of CVD in these patients.

## Methods

### Population and design

For this study, all patients enrolled in the ICD-2 trial (ISRCTN 20479861) until January 2013 were analyzed. The ICD-2 trial is an ongoing randomized controlled trial designed to evaluate the effectiveness of an implantable cardioverter defibrillator in the prevention of sudden cardiac death in dialysis patients. The study protocol has been described previously [17]. The study population consists of 55–80 year old patients with a left ventricular ejection fraction (LVEF) of at least 35%. All patients provided written informed consent and the design of the trial was approved by the local ethics committee (Leiden University Medical Center, Leiden, the Netherlands).

An extensive screening protocol was performed in all patients at the time of enrollment in the ICD-2 trial, including blood analysis, computed tomography angiography (CTA), transthoracic echocardiography (TTE) and lateral abdominal X-ray. Furthermore clinical data on demographic characteristics, coexisting conditions, use of medications and information regarding the dialysis procedures were collected. Twenty-four-hour urinary samples were collected in all patients. Residual renal function was calculated as the mean of creatinine and urea clearance adjusted for body surface area (ml/min per 1.73 m^2^). The mean of both post- and pre-dialysis plasma samples (if available) were used to estimate mean plasma creatinine and urea concentrations [18]. The residual renal function was considered zero in patients with a urinary output <100 mL/24 hours.

### Laboratory tests

After collection, the blood samples were immediately centrifuged, separated in vials and stored at -60°C for future essays. Serum levels of creatinine, calcium and phosphate were measured at time of inclusion. Concentrations of plasma c-terminal FGF-23, soluble serum Klotho (sKlotho), serum 25-hydroxyvitamin D (25(OH)D) and plasma parathyroid hormone (PTH) were measured from the frozen samples. FGF-23 was measured using an assay designed to measure C-terminal FGF-23 (Immutopics, San Clemente, CA, USA) [19]. For the measurement of sKlotho an ELISA assay was used (IBL, International GmbH, Hamburg, Germany) based on the antibodies and substrates designed, used and described by *Yamazaki* and colleagues (intra- and inter-assay coefficients of variation <5% and 8%) [20]. 25(OH)D levels were measured using a LC-MS/MS method [21].

### Echocardiography

All patients underwent 2-dimensional TTE with commercially available ultrasound equipment (M3s probe, Vivid 7, GE Vingmed, Horton, Norway). The images were digitally stored for off-line analysis (EchoPAC version 110.0.0, GE Vingmed, Horten, Norway). Using Simpson’s biplane method, left ventricular mass index (LVMI) and LVEF were assessed by a single independent observer. In accordance with the current guidelines, LV dysfunction was defined as a LVEF < 45% [22].

### Multi slice CT protocol

The CTA protocol used has been previously described [23]. Patients were scanned using a 64-slice CT scanner (Aquillion64, Toshiba Medical Systems, Otawara, Japan) or a 320-slice CT scanner (Aquilion ONE, Toshiba Medical Systems, Otawara Japan). In the presence of residual renal function, post and prehydration were performed in accordance with the nephrologist. The coronary artery calcium score (CACscore) was analyzed using the Agatston method [24]. The presence of significant coronary artery stenosis was defined as ≥50% luminal narrowing. Coronary artery disease (CAD) was defined as the presence of at least one coronary artery with stenosis. If coronary artery bypass grafting or a percutaneous coronary intervention was performed in the past, treated vessels were scored as occluded and the native vessels scored using CTA.

### Quantification of abdominal aortic calcification

The extent of aortic calcification was calculated on a lateral X-ray of the abdomen. This X-ray was taken in a standing position using standard radiographic equipment. The abdominal aorta calcification (AAC) score was calculated using a previously validated grading system, in which the extent of calcific deposits in the abdominal aorta is graded on a per segment basis [25].

### Statistical analysis

Continuous data are presented as mean ± SD and compared using the 2-tailed Student’s *t*-test. Categorical data are presented as numbers and percentages and compared using the Chi-square test. For the initial data presentation, the cohort was divided into two groups using median sKlotho as a cut-off.

The relationship between sKlotho and CVD was explored using regression models. For the AACscore, CACscore and LVMI linear regression models were used with unstandardized Beta’s and a 95% confidence interval (CI). To assist practical usability of the models, sKlotho was transformed from 1 pg/mL to 100 pg/mL, thus Beta’s represent changes of 100 pg/mL sKlotho. For the association between sKlotho and CAD as well as LV-dysfunction, logistic regression models were used. Normality, linearity and homoscedasticity of the different variables in relation to sKlotho were checked. If residuals were not normally distributed, a logarithmic transformation (ln) was performed. In both the linear and logistic regression analysis, two models were used; model 1;crude model and model 2; additionally adjusting for potential confounders: age, gender, dialysis type, dialysis vintage and residual renal function (mL/min per 1.73 m^2^).

The correlations between the different parameters in the calcium-phosphate metabolism and sKlotho levels were evaluated using a Spearman rank correlation test. In a method comparable to the one used for sKlotho, the relationship between FGF-23 and CVD (AACscore, CACscore, LVMI, CAD and LV-dysfunction) was explored using regression models adjusting for potential confounders. To assess a possible combined effect of sKlotho and FGF-23 we compared patients with a high suspected calcification risk (low sKlotho and high FGF-23) to patients with a low suspected calcification risk (high Klotho and low FGF-23) based on the median of sKlotho and FGF-23.

All statistical analyses were performed using SPSS (version 20, IBM Corp, Amonk, NY, USA). A p-value <0.05 was considered statistically significant.

## Results

### Patient characteristics

A total of 127 patients were included for the current study. The average age of the participating patients was 67 ± 7 years, 24% was female and 68% utilized hemodialysis (HD) as modality for renal replacement therapy. Median sKlotho was 460 pg/mL (25th-75th percentile; 350-620 pg/mL). The baseline characteristics are shown in Table 1. The cause of end stage renal disease (ESRD) was hypertension (24%), diabetes (18%), glomerulonephritis (9%), acute tubular necrosis (5%) and other (22%) or unknown causes (22%).

**Table 1:** **Baseline characteristics**

	Total cohort	Klotho <460 pg/mL	Klotho >460 pg/mL
	n = 127	n = 63	n = 64
Age, years	67 ± 7	67 ± 7	67 ± 8
Gender, female	30 (24%)	10 (16%)	20 (31%)*
Pulse pressure, mmHg	59 ± 24	59 ± 24	59 ± 16
Hypertension	104 (82%)	53 (87%)	51 (80%)
Diabetes	43 (34%)	24 (40%)	19 (30%)
Dialysis Modality, PD	42 (33%)	8 (13%)	34 (53%)***
Dialysis vintage, years	2.3 ± 2	2.6 ± 2.7	2.0 ± 2.2
RRF, (ml/min/1.73 m^2^)	1.8 ± 1.6	1.7 ± 1.8	2.0 ± 1.4
Anuria	30 (24%)	20 (33%)	10 (16%)*
Phosphate (mmol/L)	1.5 ± 0.4	1.6 ± 0.4	1.5 ± 0.4
Calcium (mmol/L)	2.4 ± 0.2	2.4 ± 0.2	2.4 ± 0.2
FGF-23 (RefU/mL)	7247 ± 16815	8723 ± 21627	5794 ± 10044
25(OH)D (nmol/L)	87 ± 57	102 ± 65	71 ± 42**
PTH (pmol/L)	35 ± 34	42 ± 41	28 ± 22*
Creatinine (umol/L)	659 ± 202	642 ± 211	676 ± 195
CRP (mg/L)	14.3 ± 30.7	20.1 ± 41.7	8.9 ± 12.22*
β -blocker	71 (56%)	37 (60%)	34 (54%)
ACEi	32 (25%)	19 (31%)	13 (21%)
ARB	37 (29%)	13 (21%)	24 (38%)*
Statin	79 (62%)	43 (69%)	36 (57%)
NCPB	107 (84%)	54 (87%)	53 (84%)
CCPB	50 (39%)	25 (40%)	25 (40%)
Cinacalcet	15 (12%)	7 (12%)	8 (13%)

PD; Peritoneal dialysis, RRF; Residual Renal Function, FGF-23; Fibroblast Growth Factor 23. PTH; Parathyroid hormone, ACEí; Angiotensin Converting Enzyme inhibitor, ARB; Angiotensin receptor blocker, NCPB; Non calcium containing phosphate binder, CCPB; Calcium containing phosphate binder. *;p < 0.05, **;p < 0.01; ***;p < 0.001.

After stratifying patients for plasma sKlotho levels into two groups, patients with a high sKlotho (>460 pg/mL) were more often female (31% versus 16%; p = 0.04), more often had residual renal function (84% versus 67%; p = 0.04), more frequently utilized peritoneal dialysis (PD; 53% versus 13%; p < 0.001) and more often used an angiotensin receptor blocker (ARB; 38% versus 21%; p = 0.04). Patients with a low sKlotho had a higher plasma 25(OH)D (102 nmol/L versus 71 nmol/L; p = 0.002), PTH (42 pmol/L versus 28 pmol/L; p = 0.02) and CRP (20.1 mg/L versus 8.9 mg/L; p = 0.04) (Table 1). No further differences in baseline characteristics were found between the two groups.

### sKlotho and markers for cardiovascular disease

**Figure 1:**
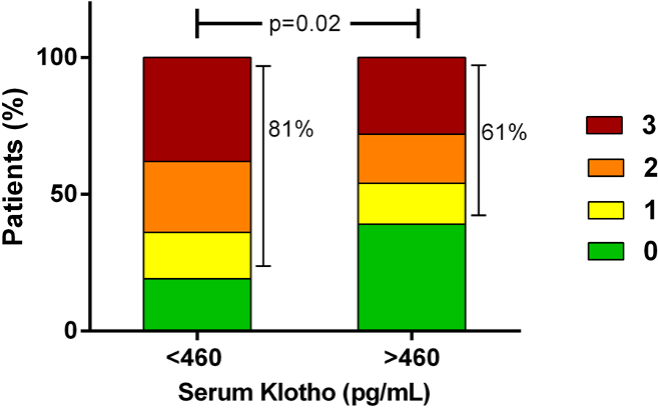
**Serum Klotho and the number of occluded coronary arteries.** Number of occluded coronary arteries in patients with low sKlotho versus patients with high sKlotho.

In the 127 included patients, the average AACscore was 9 ± 6, mean CACscore was 1134 ± 1316 and the average LVMI was 128 ± 42 g/m^2^. A total of 76 (60%) patients suffered from CAD and 13 patients (10%) had LV dysfunction. As described in the method section, only patients with LVEF ≥ 35% were included in the study. Patients with a high sKlotho (>460 pg/mL) had significantly less CAD (61% versus 81% of patients, p = 0.02; Figure 1) as well as less LV dysfunction (3% versus 19% of patients; p = 0.006). The other markers for CVD were not significantly different between both groups.

The results of the multivariate regression modelling, with the different markers for CVD as dependent variables are shown in Table 2. In model 1, the crude association between sKlotho and numerous markers for CVD was assessed. In these first models, sKlotho was not significantly associated with AACscore, CACscore, LVMI or the presence of CAD. However, a statistically significant crude association between sKlotho and the presence of LV-dysfunction was observed (OR: 0.58; 95% CI 0.37-0.92; p = 0.02). In model 2 the association between sKlotho and CVD was assessed after adjusting for confounders (age, gender, (ln)dialysis vintage, dialysis type and residual renal function). After the adjustments made in model 2, sKlotho showed no significant association with any of these investigated markers for CVD.

**Table 2:** **Association between sKlotho and cardiovascular disease**

***AACscore***	***Beta***	***CI***	***p-value***
Model 1. Crude association	0.33	-0.26–0.91	0.27
Model 2. (+ Confounders)*	0.58	-0.07–1.22	0.08
***CACscore***	***Beta***	***CI***	***p-value***
Model 1. Crude association	-0.06	-0.31–0.20	0.66
Model 2. (+Confounders)*	0.08	-0.19–0.36	0.55
***LVMI***	***Beta***	***CI***	***p-value***
Model 1. Crude association	-1.27	-5.58–3.04	0.56
Model 2. (+Confounders)*	1.55	-3.49–6.60	0.54
***LV-dysfunction (LVEF < 45%)***	***OR***	***CI***	***p-value***
Model 1. Crude association	0.58	0.37–0.92	0.02
Model 2. (+Confounders)*	0.66	0.39–1.11	0.12
***CAD (any vessel occluded)***	***OR***	***CI***	***p-value***
Model 1. Crude association	0.82	0.65–1.05	0.11
Model 2. (+Confounders)*	0.91	0.66–1.23	0.52

Beta’s represent changes of 100 pg/mL in sKlotho.

AACscore; Abdominal aorta calcification score, CACscore; Coronary artery calcification score, LVMI; Left ventricular mass index, LV-dysfunction; Left ventricular dysfunction. CAD; Coronary artery disease.

**Model 1**; sKlotho. *Model 2; + age, gender, (ln)dialysis vintage, dialysis type, residual renal function.

### sKlotho and markers of the calcium-phosphate axis

The associations between the different parameters in the calcium-phosphate axis are shown in Table 3. Serum sKlotho level was significantly associated with a lower plasma 25(OH)D (r = -0.22, p = 0.01) and lower PTH (r = -0.26; p < 0.01). FGF-23 showed a strong positive association with phosphate level (r = 0.51; p < 0.001) and PTH r = 0.30; p < 0.001). There was no crude association between serum FGF-23, PTH, 25(OH)D or phosphate and any of the investigated markers for CVD (data not shown). Furthermore, in the different multivariate regression models, FGF-23 was not independently associated with CVD after adjusting for confounders (Although a LVEF < 45% showed a trend toward an independent association with FGF-23, OR: 0.51 (0.26-1.00; p = 0.053, Table 4).

**Table 3:** **Spearman correlation coefficients of parameters of calcium-phosphate metabolism and sKlotho levels**

	***Phosphate***	***25(OH)D***	***PTH***	***FGF-23***	***sKlotho***
	(mmol/L)	(nmol/L)	(pmol/L)	(RefU/L)	(pg/mL)
***25(OH)D***	r = 0.06				
(nmol/L)	p = 0.53				
***PTH***	r = 0.30	r = -0.01			
(pmol/L)	p < 0.01	p = 0.90			
***FGF-23***	r = 0.51	r = -0.03	r = 0.29		
(RefU/L)	p < 0.001	p = 0.76	P = 0.001		
***sKlotho***	r = -0.04	r = -0.22	r = -0.26	r = 0.11	
(pg/mL)	p = 0.64	p = 0.01	p < 0.01	p = 0.22	
***Calcium***	r = 0.04	r = 0.05	r = -0.09	r = 0.22	r = -0.04
(mmol/L)	p = 0.67	p = 0.55	p = 0.34	p = 0.01	p = 0.66

PTH; Parathyroid hormone, FGF-23; Fibroblast Growth Factor 23. Indicated are correlation coefficients (r) with levels of significance (p).

**Table 4:** **Association between FGF-23 and cardiovascular disease**

***AACscore***	***Beta***	***CI***	***p-value***
Model 1. Crude association	0.72	-0.13-1.57	0.10
Model 2. (+ Confounders)^*^	0.49	-0.35-1.34	0.25
***CACscore***	***Beta***	***CI***	***p-value***
Model 1. Crude association	0.25	-0.13-0.63	0.20
Model 2. (+Confounders)^*^	0.001	-0.40-0.40	1.00
***LVMI***	***Beta***	***CI***	***p-value***
Model 1. Crude association	0.48	-5.79-6.75	0.88
Model 2. (+Confounders)^*^	-0.70	-7.40-6.00	0.84
***LV-dysfunction (LVEF < 45%)***	***OR***	***CI***	***p-value***
Model 1. Crude association	0.68	0.40-1.16	0.16
Model 2. (+Confounders)^*^	0.51	0.26-1.01	0.053
***CAD (any vessel occluded)***	***OR***	***CI***	***p-value***
Model 1. Crude association	0.94	0.66-0.63	0.20
Model 2. (+Confounders)^*^	0.65	0.41-1.04	0.08

Beta’s represent changes in LnFGF-23.

AACscore; Abdominal aorta calcification score, CACscore; Coronary artery calcification score, LVMI; Left ventricular mass index, LV-dysfunction; Left ventricular dysfunction. CAD; Coronary artery disease.

**Model 1**; sKlotho. *Model 2; + age, gender, (ln)dialysis vintage, dialysis type, residual urine production.

A total of 26 patients had a high median serum FGF-23 (>2625 RefU/L) combined with a low sKlotho (<460 pg/mL), compared to 26 patients with a low FGF-23 (<2625 RefU/L) and high sKlotho level (>460 pg/mL). Patients in the former group had a significantly higher CACscore (1242 ± 1037 versus 485 ± 656; p = 0.01) as well as a trend towards lower LVEF (52 ± 7% versus 55 ± 6%; p = 0.07) and more CAD (85% versus 60%; p = 0.07).

## Discussion

Recently, sKlotho has emerged as a powerful player in calcium-phosphate homeostasis that is thought to contribute to the high burden of CVD in CKD patients [4]. To the best of our knowledge, the present study is the first study that investigated the association between sKlotho and CVD in dialysis patients. Contrary to our hypothesis, while patients with a low sKlotho demonstrated LV dysfunction and CAD more often, there was no independent association between sKlotho and CVD in the present cohort.

### sKlotho and cardiovascular disease

Several clinical studies have suggested that Klotho exerts strong cardioprotective effects. For instance, Klotho has been shown to protect against vascular calcifications in rodent models of CKD while in humans without CKD, higher sKlotho levels have been related to a lower incidence of mortality and CVD [8, 9, 26, 27]. Moreover, low sKlotho levels have been associated with increased arterial stiffness in CKD patients [28]. Further support for a direct role of Klotho in vascular homeostasis comes from *in vitro* studies showing endogenous expression of Klotho in human vascular smooth muscle cells (VSMCs) [14]. Interestingly, inhibition of sKlotho expression in aortic VSMCs resulted in accelerated calcification of these cells [14]. However, the exact role of Klotho in the progression of CVD in dialysis patients remains to be elucidated.

In the present study, dialysis patients with a low sKlotho demonstrated LV-dysfunction and CAD more frequently. However, an independent association between sKlotho and CVD was not observed. There are several possible explanations for these findings.

First, disorders of mineral homeostasis, as well as CVD start to develop in the early stages of CKD [1, 3]. Therefore, patients with ESRD have been exposed to an environment predisposing to vascular calcifications for a prolonged period of time. In our study, we solely measured sKlotho levels at time of inclusion and as a consequence, the sKlotho levels in our study do not reflect the total ‘burden’ of Klotho deficiency in the dialysis patients’ preceding years with CKD. Thus, the association between sKlotho and vascular pathology might be diminished by the time patients have developed ESRD.

Second, the role of sKlotho in the development of atherosclerotic disease in dialysis patients might be overshadowed by the large amount of other pathophysiological stimuli for CVD prevalent in these patients, such as smoking, obesity, diabetes and hypertension among others. This might explain the disparity between data found in rodents with only Klotho deficiency and patients suffering from a wide variety of co-morbidities [8, 9].

A third explanation could be that sKlotho simply has no independent contribution to CVD in dialysis patients. Several very recent publications revealed similar results [29–32]. In patients with CKD up to stage IV, sKlotho was not associated with AACscore nor was it predictive for cardiovascular outcomes [29, 30]. In addition, pre-clinical studies have provided for a limited role of Klotho in the vasculature since they showed a very low or absent vascular Klotho expression and Klotho mediated effects in human and murine VSMCs [31, 32].

Even though sKlotho does not appear to be independently associated with CVD in dialysis patients in the present study, CAD and LV dysfunction was more often present in patients with a low sKlotho level. Furthermore, patients with a combination of low serum sKlotho and high FGF-23 showed a significantly higher CACscore as well as a trend toward a lower LVEF and less CAD. Therefore, sKlotho might be an effective biomarker for CVD. In this respect, it would be interesting to explore the association between urinary sKlotho and CVD, since recent studies have illustrated that urinary sKlotho might correlate better with renal function that plasma sKlotho [33].

### Effect of treatment modalities on sKlotho level

In the investigated cohort two interesting observations were made regarding the association between treatment modalities and sKlotho levels. In line with a previous study that showed that losartan increased sKlotho levels, we observed that patients with a higher sKlotho more often used an ARB [34]. It was observed in rats that administration of angiotensin-II reduced Klotho gene expression at both mRNA and protein level in the kidney, whereas ARB and renin inhibitors increased Klotho expression [35, 36]. These studies suggest a direct inhibitory effect of the renin-angiotensin-aldosterone system (RAAS) on Klotho expression [37].

In addition, we observed that patients with a higher sKlotho more often performed PD. This is an intriguing difference, since sKlotho is excreted in the peritoneal dialysate (median amount of 114 ng/day), while the molecular weight of sKlotho is too high (120–130 kDa) to allow clearance by hemodialysis [38]. A possible explanation could be that the higher residual renal function in PD patients as observed in our cohort, reflects a healthier kidney and thus higher sKlotho levels.

### Limitations

This study has some limitations. Data on dietary phosphate intake was not collected, which is a known determinant for both serum phosphate and FGF-23 levels. Furthermore sKlotho was not measured at a fixed time and thus a circadian variation in the measured sKlotho, as described in previous studies, cannot be excluded [39]. However, all blood samples in the ICD-2 trial were taken in the morning, somewhat decreasing this variability.

## Conclusions

In the present cohort of dialysis patients, sKlotho level was not independently associated with cardiovascular disease. However, patients with a low sKlotho level (<460 pg/mL) did show CAD and LV dysfunction more often. Therefore, while sKlotho might be a marker for CVD in dialysis patients, the current data does not support a direct cardioprotective effect of sKlotho.

## Abbreviations

25(OH)D: 25-hydroxyvitamin D
AACscore: Abdominal Aorta Calcification score
ARB: Angiotensin Receptor Blocker
CACscore: Coronary Artery Calcification score
CAD: Coronary Artery Disease
CKD: Chronic Kidney Disease
CVD: Cardiovascular Disease
CTA: Computed Tomography Angiography
ESRD: End Stage Renal Disease
FGF-23: Fibroblast Growth Factor 23
HD: Hemodialysis
LV: Left Ventricular
LVEF: Left Ventricular Ejection Fraction
LVMI: Left Ventricular Mass Index
PD: Peritoneal Dialysis
PTH: Parathyroid Hormone
sKlotho: Serum Klotho
TTE: Transthoracic Echocardiography
VSM: Vascular Smooth Muscle Cells.
